# Use of RNAlater in fluorescence-activated cell sorting (FACS) reduces the fluorescence from GFP but not from DsRed

**DOI:** 10.1186/1756-0500-3-328

**Published:** 2010-12-06

**Authors:** Ismail Zaitoun, Christopher S Erickson, Kathy Schell, Miles L Epstein

**Affiliations:** 1 Department of Anatomy, University of Wisconsin School of Medicine and Public Health, 1300 University Avenue, Madison, WI 53706, USA; 2 Flow Cytometry Facility, Paul P. Carbone Comprehensive Cancer Center, 1111 Highland Ave., Madison WI 53706, USA

## Abstract

**Background:**

Flow cytometry utilizes signals from fluorescent markers to separate targeted cell populations for gene expression studies. However, the stress of the FACS process could change normal gene expression profiles. RNAlater could be used to stop such changes in original gene expression profiles through its ability to denature RNase and other proteins. The normal conformational structure of fluorescent proteins must be maintained in order to fluoresce. Whether or not RNAlater would affect signals from different types of intrinsic fluorescent proteins is crucial to its use in flow cytometry; this question has not been investigated in detail.

**Findings:**

To address this question, we analyzed the effect of RNAlater on fluorescence intensity of GFP, YFP, DsRed and small fluorescent molecules attached to secondary antibodies (Cy2 and Texas-Red) when used in flow cytometry. FACS results were confirmed with fluorescence microscopy. Our results showed that exposure of YFP and GFP containing cells to RNAlater reduces the intensity of their fluorescence to such an extent that separation of such labeled cells is difficult if not impossible. In contrast, signals from DsRed2, Cy2 and Texas-Red were not affected by RNAlater treatment. In addition, the background fluorescence and clumping of dissociated cells are altered by RNAlater treatment.

**Conclusions:**

When considering gene expression studies using cell sorting with RNAlater, DsRed is the fluorescent protein of choice while GFP/YFP have severe limitations because of their reduced fluorescence. It is necessary to examine the effects of RNAlater on signals from fluorescent markers and the physical properties (e.g., clumping) of the cells before considering its use in cell sorting.

## Background

Fluorescent labeling enables researchers to trace optically a particular population of cells *in vitro *or *in vivo*. FACS procedure is used to separate targeted populations for further biochemical characterization and in particular to permit isolation of intact mRNA for microarray and quantitative real time PCR studies. However, sorted cells go through a series of steps that could induce stress and change gene expression. Mechanical force has been shown to modulate global gene expression and signaling pathways in different cell types [1,2]. Such force is typically used in dissociating cells. The hydrodynamic forces utilized in the operation of the FACS could affect cell viability as well. Indeed, several reports have shown a significant decrease in viability in different cell types after sorting by flow cytometry [3-7]. We observed a reduction of ~10% in the viability of sorted cells, which is consistent with these reports. While FACS is an efficient method for isolating cells for gene expression analysis, it is essential to prevent changes in normal global gene expression of sorted cells, a result which can be effected by treating cells with RNAlater. RNAlater preserves the product of normal gene expression by denaturing RNase and other cellular proteins, thus maintaining RNA integrity for gene expression studies using both microarray and quantitative real time PCR [8,9]. RNAlater contains ammonium sulfate salt solutions, which have the ability to denature RNase at a controlled pH [10,11]. However, if cells are prepared in RNAlater prior to sorting, the conformational structure of fluorescent proteins must be maintained within certain limits in order to fluoresce [12-14]. Because of its ability to denature protein, we investigated whether RNAlater would affect signals from fluorescent markers, such as GFP, YFP, DsRed, Cy2 and Texas-Red.

## Results and Discussion

**Figure 1:**
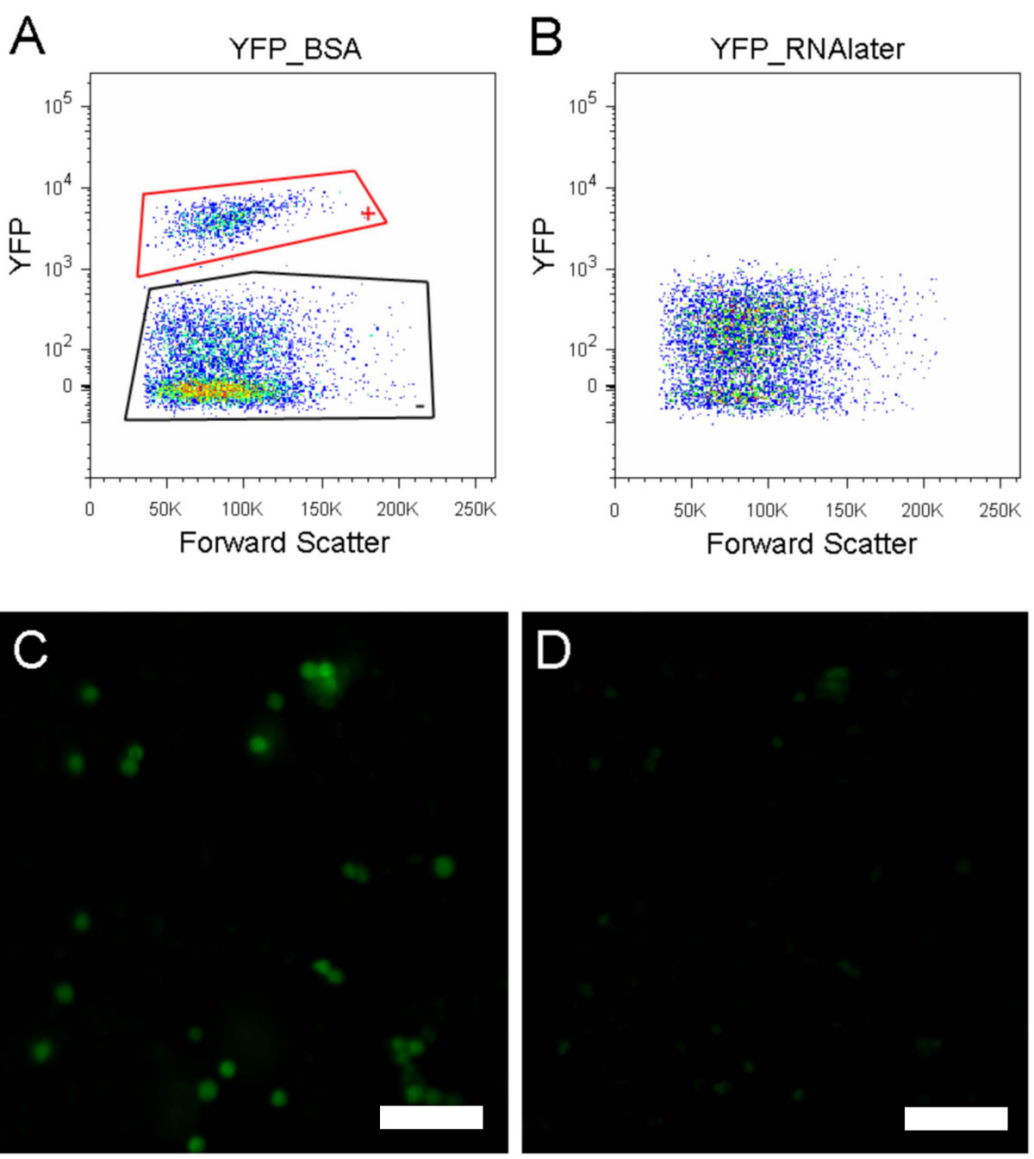
**RNAlater decreases fluorescence of YFP**. (A & B) FACS results. All data in FACS figures are restricted to cells defined by forward and side light scatter and further to singlet events. The YFP positive cells are shown enclosed by red lines and negative cells are shown enclosed by black lines. (A) Cells in BSA: a population of YFP positive cells is clearly distinguished from YFP negative cells. (B) Cells in RNAlater: the two populations are not discernable. (C & D) dissociated cells were observed under the fluorescence microscope (C) YFP positive cells in BSA. (D) YFP positive cells one minute after addition of RNAlater. The intensity of the YFP decreased substantially in the presence of RNAlater. (C & D) Exposure time: 200ms; scale bar: 50 microns.

Green fluorescent protein (GFP), originally isolated from jellyfish *Aequorea victoria*, is a soluble globular protein with a chromophore in its center that emits green fluorescence (for review see Ref. [15]). The native structure of the GFP and its variants (YFP) consists of a chromophore surrounded by 11 beta sheets and capping alpha helices [16-18]. Protein denaturation exposes the chromophore to water, an event which results in quenching of fluorescence [12]. To investigate the effect of RNAlater on YFP fluorescence, we obtained tissue from transgenic mice that express YFP in neural crest-derived cells [19]. The dissociated cells were sorted on a flow cytometer (FACS Vantage SE, BD Bioscience, San Jose, California) into YFP positive and negative populations. These populations were clearly separable and distinct when initially suspended in BSA (Figure 1A). In contrast, YFP positive and negative populations were not distinct when initially treated with RNAlater (Figure 1B); YFP fluorescence decreased 94% (Figures 1A and 1B). When dissociated YFP positive cells were examined under the microscope, the YFP signal was extinguished in less than a minute after addition of RNAlater (Figure 1C,1D). The RNAlater treatment also increased the background fluorescence of the YFP negative cells and the forward scatter of both YFP positive and negative cells. It is likely that these changes in scatter, a crude measure of cell size, reflect changes in the optical properties of RNAlater-treated specimens. Similar increases in the background fluorescence of RNAlater treated specimens has been observed by other groups [9,20]. We also studied the effects of RNAlater on a GFP positive T cell line [21]. The median fluorescence intensity of the T cells treated with RNAlater declined by 80% as measured by flow cytometry. Cellular proteins differ widely in their conformational stabilities, and studies have shown that the fluorescence of GFP and its variants is pH dependent [13]. It has been reported that 80% of GFP fluorescence is lost at pH 6.5 and lower [13]. Indeed, we found that the pH of RNAlater is 5.6. We attribute the quenching of the GFP and YFP monomeric proteins to the acid pH found in RNAlater. Rosenberg et al. (2003) found a reduction of similar magnitude (80%) of GFP fluorescence after RNAlater treatment but did not show whether GFP+ cells could still be distinguished from GFP negative cells by flow cytometry. Our data show that exposure of YFP and GFP containing cells to RNAlater reduces the amount of their fluorescence to such an extent that separation is difficult, or not possible.

**Figure 2:**
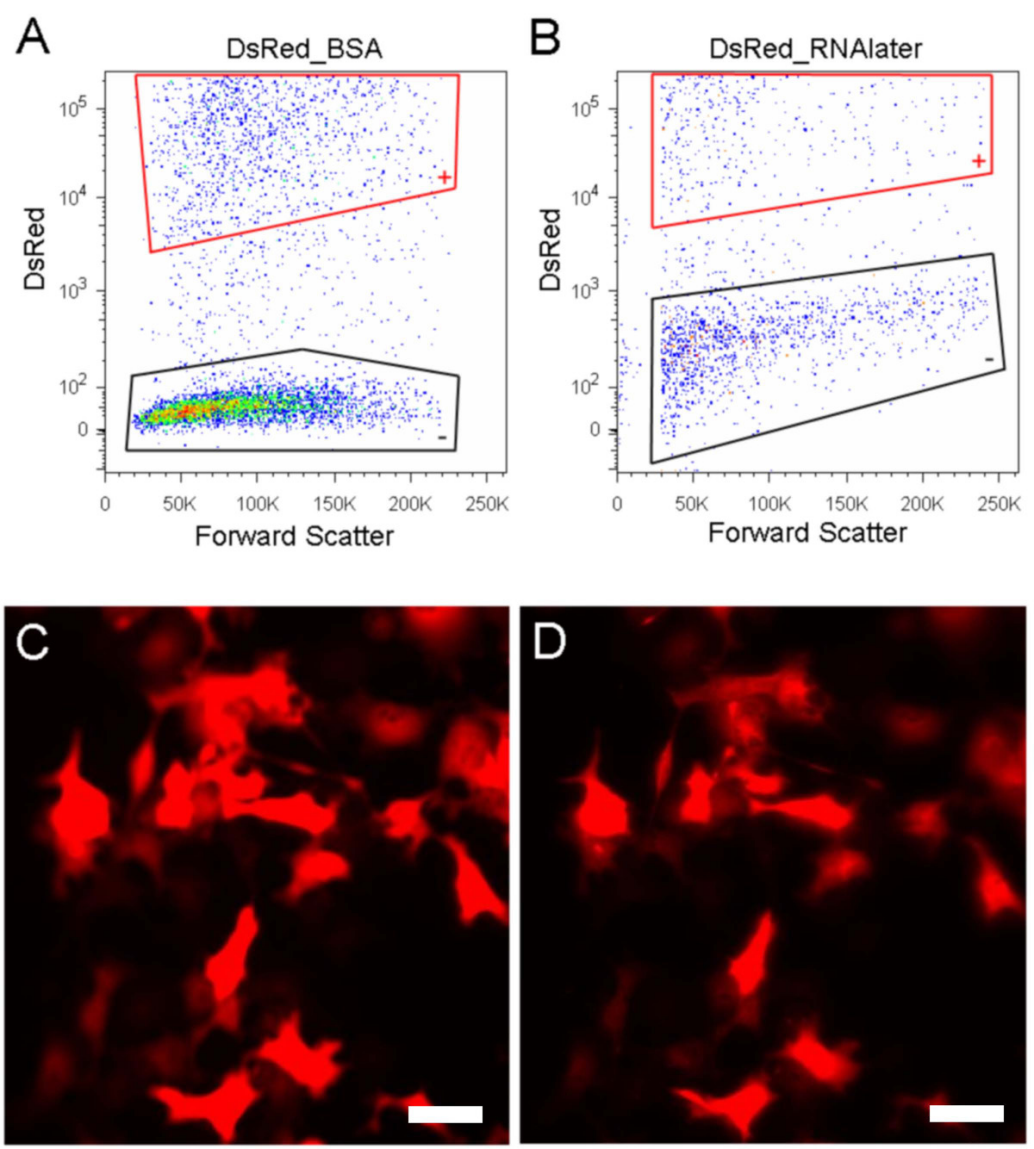
**Fluorescence of DsRed2 protein is not affected in RNAlater**. (A & B) FACS results. All data in FACS figures are restricted to single cells defined by forward and side light scatter; clumped and ruptured cells (debris) are not displayed in the figure. DsRed positive and negative COS-7 cells were mixed before flow cytometry sorting. (A) Cells in BSA: a population of DsRed2 positive cells is clearly distinguished from DsRed2 negative cells. The cells with intermediate fluorescence intensity between the positive and negative populations represent newly dividing cells, which are in the initial stages of DsRed expression. (B) Cells in RNAlater: fewer cells are shown here than in (A) because RNAlater has induced cell clumping, so fewer singlets are available to the sorter. RNAlater did not quench fluorescent signals from analyzed DsRed positive cells. (C & D) Dissociated cells were observed under the fluorescence microscope (C) DsRed2 positive cells in BSA. (D) DsRed2 positive cells after addition of RNAlater. The intensity of the DsRed2 was stable in the presence of RNAlater. (C & D) Exposure time: 30ms; scale bar: 50 microns.

While RNAlater reduces the fluorescence of GFP and YFP, it does not reduce the signal from the red fluorescent protein (DsRed2; Figure 2). DsRed originally isolated from the *Discosoma *genus, is a commonly used tetrameric fluorescent protein (for review see Ref. [22]). It has a beta-can structure similar to monomeric GFP but with a different chromophore [23]. While the mature form of tetrameric DsRed emits a red fluorescence, its immature monomer emits a green fluorescence during the transition to the formation of its tetrameric structure [24]. DsRed2 is a mutant of DsRed, with a shorter maturation time [25]. To evaluate the effect of RNAlater on DsRed2, COS-7 cells were electroporated with the DsRed2 vector, cultured, and resuspended under standard conditions. DsRed positive and negative COS-7 cells were mixed before sorting by flow cytometry. In contrast to its effect on GFP and YFP, we found no detectable change in the intensity of DsRed2 fluorescence after RNAlater treatment (Figure 2A, B). Experiments were repeated with HEK 293T cells and similar results were obtained (data not shown). Both COS-7 and HEK 293 cells showed a large increase in cells clumping after RNAlater treatment, thereby reducing the number of single cells available for sorting. Observations with the fluorescence microscope also showed no decrease in DsRed2 fluorescence (Figure 2C, D). In contrast to GFP, the fluorescence of DsRed is stable within a wide range of pH 5.0-12.0 [22,26,27], a finding that suggests why RNAlater (pH 5.6) does not quench the DsRed2 fluorescence. However, after bringing the pH of the RNAlater down to 4.0, red fluorescence from mature DsRed2 was substantially reduced as viewed under the fluorescence microscope, but green fluorescence from immature monomers of DsRed2 was still visible. We conclude that RNAlater does not quench fluorescent signals from tetrameric DsRed2 protein.

Our findings indicate that RNAlater treatment reduces GFP and YFP fluorescence, making separation of fluorescent and non-fluorescent cells difficult or impossible. Rosenberg et al. (2003) found a similar decrease in GFP fluorescence but they did not attempt to separate the two populations using flow cytometry. In addition, their results in tissues indicated that fluorescence from both GFP and DsRed was stable over an extended period of time. In contrast, we show that GFP fluorescence was extinguished in minutes, while DsRed fluorescence was stable. Their experiments were conducted using a GFP expressing cell line that was transfected with DsRed. One possible explanation for this difference in stability of fluorescence in tissues is that they might have detected the green fluorescence from the monomeric immature species of DsRed protein [24], and not from GFP itself. Indeed, we found that the green fluorescence from immature species of DsRed2 to be more resistant to quenching by RNAlater (pH 4.0) than red fluorescence from mature DsRed2.

It is noteworthy that RNAlater affects the physical properties of treated cells. Because of their large size, the changes (clumping) in the COS-7 and HEK 293 cells were more apparent than changes in primary cells. As a result, the sorter recognized the majority of RNAlater-treated COS-7 and HEK 293 cells as debris, while a large proportion of primary cells remained as singlets and were sorted normally.

**Figure 3:**
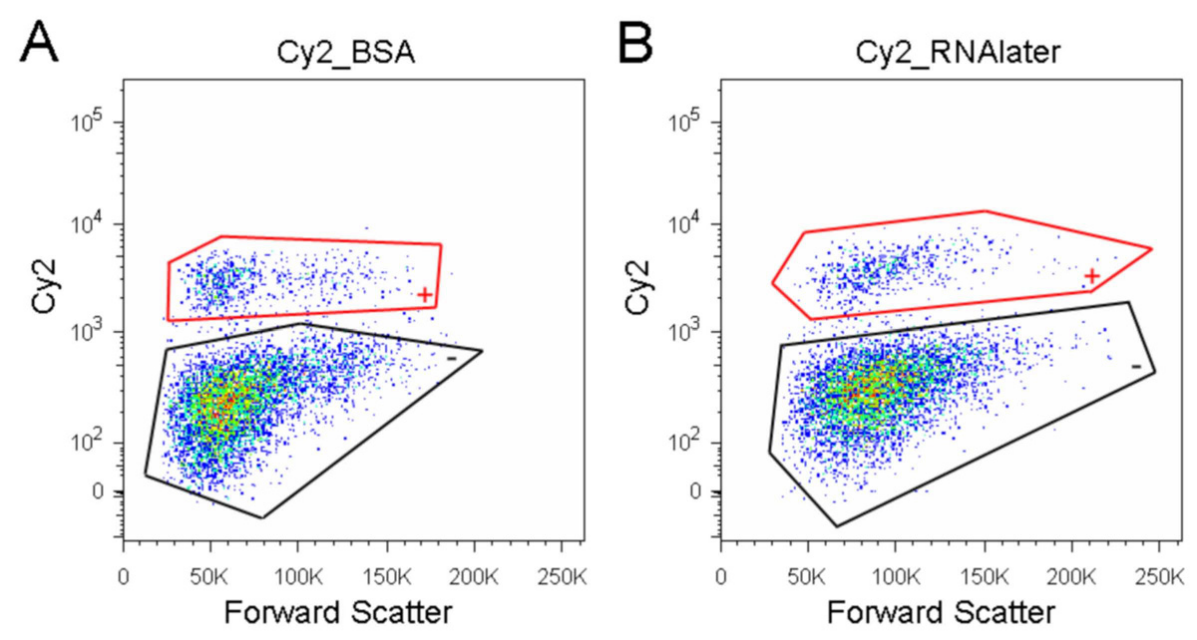
**Fluorescence of Cy2 is not affected in RNAlater**. (A & B) FACS results. All data in FACS figures are restricted to cells defined by forward and side light scatter and further to singlet events. Dissociated cells were fixed and immunostained. The immunostained cells were visualized with secondary antibody conjugated with Cy2. The Cy2 positive cells are shown enclosed by red lines and negative cells are shown enclosed by black lines. (A) Cells in BSA: a population of Cy2 positive cells is clearly distinguished from Cy2 negative cells. (B) Cells in RNAlater: the two populations are distinguishable as well.

Small fluorescent molecules are markers used to stain specific cell types extrinsically. For example, immunofluorescence is achieved by using antibodies conjugated to these molecules. We immunostained fixed cells that were visualized with secondary antibody conjugated with Cy2. These immunostained cells were treated with either BSA or RNAlater and sorted by flow cytometry. We observed no difference in the Cy2 labeled populations (Figure 3A, B). We repeated these experiments on tissues labeled with Texas-Red conjugated antibody and observed no decrease in fluorescence. Similar results were obtained using a fluorescence microscope (data not shown). We conclude that RNAlater does not affect the signal from small fluorescent molecules attached to secondary antibodies, an observation that is consistent with a previous report [9].

In summary, we report that RNAlater diminishes the intensity of the fluorescent proteins GFP and YFP, and hinders their utility for sorting by flow cytometry. In contrast, RNAlater does not diminish the fluorescence of DsRed2 protein and the small molecule fluorophores Cy2 and Texas-Red. These results suggest that targeted DsRed2 expression in mice should be the choice for gene expression studies when RNAlater is used. However, it should be noted that RNAlater affected the physical properties (clumping) of the cells and their ability to be sorted regardless of the fluorescent protein expressed. Thus, it is necessary to examine the effects of RNAlater on signals from fluorescent markers and the physical properties of the cells before considering its use in cell sorting.

## Methods

### Animal tissues

All procedures were approved by the Institutional Animal Care and Use Committee of the University of Wisconsin-Madison. We obtained tissues from transgenic mice that express YFP in neural crest-derived cells [19]. Pregnant mice were anesthetized with isoflurane vapor, sacrificed by cervical dislocation, and fetuses were removed at E14.5. YFP positive fetuses were identified under the fluorescent microscope and their gastrointestinal tracts were harvested and pooled. Collected tissues were dissociated in a mixture of 3 mg/ml collagenase, 1 mg/ml Dispace, 1 mg/ml BSA, and 0.5 mg/ml DNAase for 20 minutes at 37°C, washed in PBS, and triturated. The dissociated cells were resuspended in either 0.1% bovine serum albumin in PBS (BSA) or RNAlater (Qiagen, Hilden, Germany, final concentration ~50% in BSA), and kept on ice for 0.5-1 hour before FACS sorting.

### Immunostaining

For whole mount staining, paraformaldehyde-fixed tissues were incubated with human anti-Hu (Epstein laboratory, Madison, WI), followed by goat anti-human-Texas Red (Jackson ImmunoResearch, West Grove, PA). Fixed dissociated cells were incubated with chicken anti-GFP (Aves, Tigard, Or) for 2 hours at room temperature, washed in PBS, incubated in donkey anti-chicken-CY2 (Jackson ImmunoResearch) for 2 hours, washed in PBS, and sorted on the flow cytometer as described below.

### Cell culture and DsRed2 transfection

The GFP positive T cell line was cultured as described [21]. HEK-293 cells were cultured in Eagle's minimum essential medium (Fisher Scientific, Pittsburg, PA) with 10% fetal bovine serum, 1% penicillin/streptomycin, 1% L-glutamine, 1% sodium pyruvate, and 400 μg/ml gentamicin. COS-7 cells were cultured in Dulbecco's modified Eagle's medium (Fisher Scientific) with 10% cosmic calf serum and 1% penicillin/streptomycin. Both cell types were cultured in 37°C 5% CO2-air atmosphere. HEK-293 cells and COS-7 cells were transfected with DsRed2 plasmid by electroporation as described previously [28]. After 3-5 days of transfection, both lines were dissociated with trypsin into single cells and kept on ice for 0.5-1 hour. All three cell lines were sorted on the flow cytometer as described below.

### Flow Cytometry

Cell suspensions were prepared as described above. 0.25 ml of dissociated cells in BSA was filtered through 20 um Nitex into tubes containing either 1.0 ml RNALater or 1.0 ml BSA. Filtered cells were separated into fluorescent positive and negative cells by a flow cytometry (FACS Vantage SE, BD Bioscience, San Jose, California).

## Competing interests

The authors declare that they have no competing interests.

## Authors' contributions

IZ and MLE, designed the research, performed the experiments, analyzed the results, and drafted the manuscript. CSE, performed the experiments and analyzed the results. KS**, **carried out the flow cytometry experiments and commented on the manuscript. All authors read and approved the final manuscripts.
